# Comparative Evaluation of the Radical-Scavenging Activities of Fucoxanthin and Its Stereoisomers

**DOI:** 10.3390/molecules19022100

**Published:** 2014-02-17

**Authors:** Yiping Zhang, Hua Fang, Quanling Xie, Jipeng Sun, Rui Liu, Zhuan Hong, Ruizao Yi, Hao Wu

**Affiliations:** 1 Nanjing University of Chinese Medicine, Nanjing 210023, Jiangsu, China; E-Mails: zhangyiping310@163.com (Y.Z.); cpulr@126.com (R.L.); 2 State Oceanic Administration Third Institute of Oceanography, Xiamen 361005, Fujian, China; E-Mails: fangh6115@163.com (H.F.); XieQuanling@163.com (Q.X.); sunjipeng80@163.com (J.S.); xm_hzh@163.com (Z.H.)

**Keywords:** fucoxanthin, stereoisomers, radical-scavenging activities

## Abstract

Fucoxanthin (Fuco) is a characteristic carotenoid of brown seaweeds. In the present study, Fuco and its stereoisomers 9'*Z*-Fuco, 13*Z*- and 13'*Z*-Fuco were extracted from *Laminaria japonica Aresch*. They were isolated and purified by silica gel column chromatography, Sephadex LH-20, and reversed-phase HPLC. The radical-scavenging activities of the three stereoisomers were evaluated toward 1,1-diphenyl-2-picrylhydrazyl (DPPH) radical, 2-2'-azinobis-(3-ethylbenzothiazoline-6-sulfonic acid) (ABTS) radical, hydroxyl radical, and superoxide radical. The order of 1,1-diphenyl-2-picrylhydrazyl (DPPH) radical-scavenging activity was 13*Z*- and 13'*Z*-Fuco > (all-*E*)-Fuco > 9'*Z*-Fuco. The order of 2-2'-azinobis-(3-ethylbenzothiazoline-6-sulfonic acid) (ABTS) and hydroxyl radical-scavenging activities were 9'*Z*-Fuco > (all-*E*)-Fuco > 13*Z*-and 13'*Z*-Fuco. The order of superoxide radical-scavenging activity was 13*Z*- and 13'*Z*-Fuco > (all-*E*)-Fuco > 9'*Z*-Fuco. The scavenging activities of Fuco and its stereoisomers toward the four radical types were all dose-dependent. The ABTS, DPPH, and superoxide radical-scavenging activities were all weaker than that of tocopherol (VE), while their hydroxyl radical-scavenging activities were stronger than that of VE. The results confirmed that Fuco and its stereoisomers have potent antioxidant activities.

## 1. Introduction

Reactive oxygen species (ROS), including superoxide anion radical (O_2_^•−^), hydroxyl radical (OH^•−^), and H_2_O_2_ are formed during normal metabolic processes, and can easily initiate the peroxidation of membrane lipids and thus lead to accumulation of lipid peroxides [1]. Free radicals are produced continuously in all cells as part of normal cellular function, but excessive production of free radicals originating from endogenous sources can play key roles in many diseases [2]. Free radical scavengers and antioxidants can reduce the extent of lipid peroxidation and the generation of ROS. Based on the understanding of antioxidant mechanisms, the critical importance of antioxidants for human health has been recognized. Furthermore, because of their minimal toxic effects, antioxidants derived from natural sources, have been widely used instead of synthetic antioxidants to increase the shelf-life of foods [3,4].

One of the important characteristics of carotenoids is their ability to scavenge ROS, thus protecting cells and tissues from the damaging effects of ROS. The free radicals and singlet oxygen produced in the body by normal aerobic metabolism can react with various components of living cells and cause structural changes leading to diseases such as accelerated ageing, atherogenesis, ischemia, infant retinopathy, and carcinogenesis. Carotenoids are known to help protect against diseases and age-related phenomena caused by oxidants [5]. Isomerization is a common feature of carotenoids, such as fuxoxanthin (Fuco), because of the presence of conjugated double bonds [6]. Generally speaking, the *E*-isomers of carotenoids are more common in foods and more stable compared with their *Z*-isomer counterparts. Recently, the biological function of *Z*- and *E*-lycopenes has been studied [7,8]. However, the biological significance of geometrical isomers of other carotenoids such as Fuco has received little attention.

**Figure 1:**
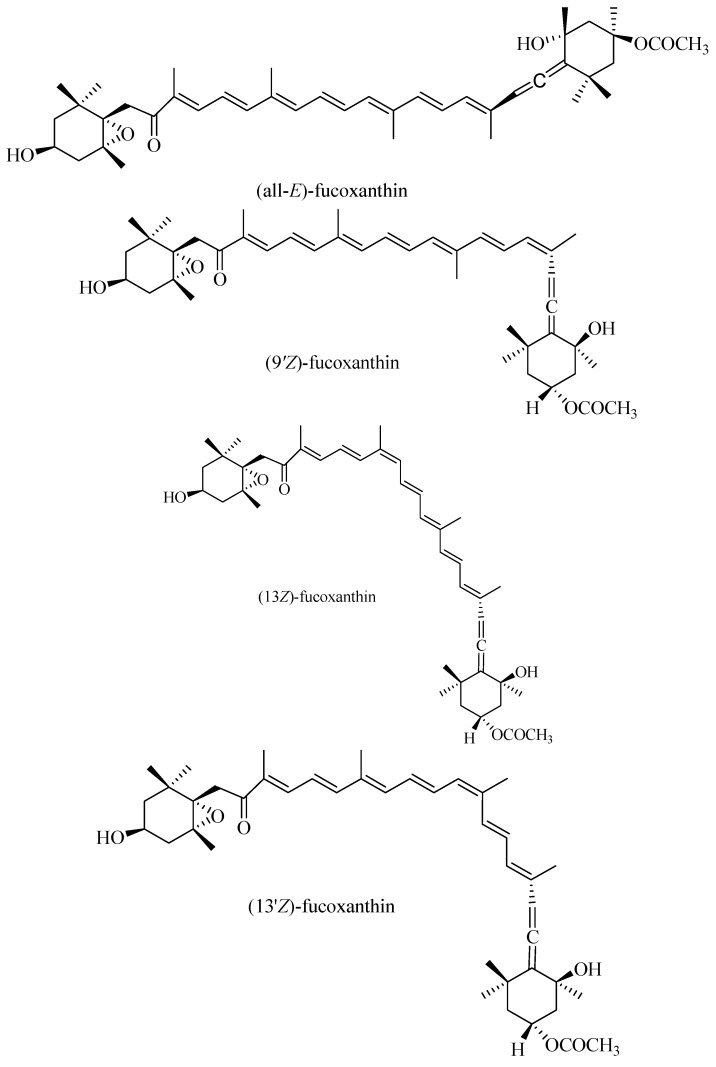
Structures of Fuco isomers.

Fuco is the most abundant compound among all carotenoids and is mainly found in brown seaweeds, accounting for more than 10% (*w*/*w*) of the carotenoid content [9]. Fuco possesses an unusual allenic bond and a 5,6-monoepoxide which makes its structure unique (Figure 1). It was reported that Fuco can induce apoptosis in leukemia HL-60 cells [10], carcinoma HepG2 cells [11], and hepatoma cells [12]. It has also been shown that Fuco has radical-scavenging efficacy [13] and exerts a protective effect against H_2_O_2_ or UV-B induced cell damage [14,15]. The growth inhibitory effects of the *Z*- and *E*-isomers of Fuco on HL-60 cells and Caco-2 cells have been reported previously [7]. To the best of our knowledge, the differences in radical-scavenging activity between Fuco *Z*- and *E*-isomers are still unclear. Therefore, in the present study, the activities of Fuco *Z*- and *E*-isomers against different radicals were investigated to elucidate the relationship between their biological effects and ROS scavenging abilities.

## 2. Results and Discussion

### 2.1. Separation and Purification of Fuco Stereoisomers

**Figure 2:**
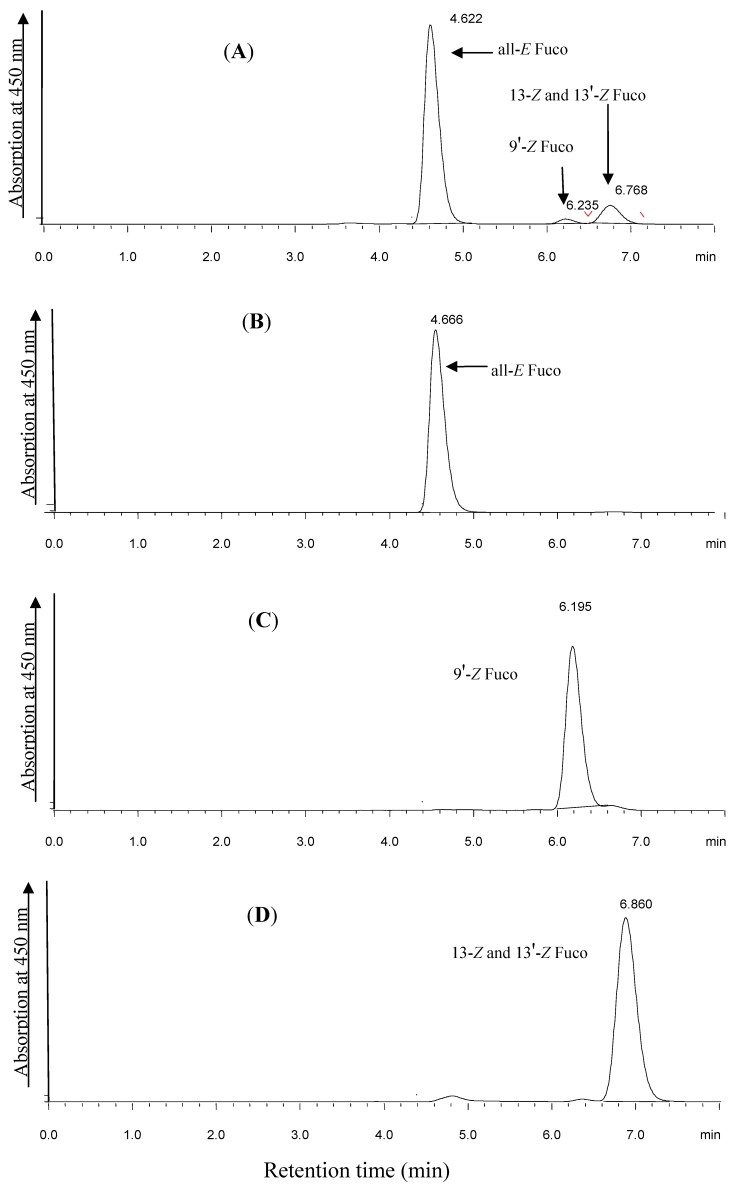
Chromatograms of Fuco and its isomers. (**A**) Crude product; (**B**) All-*E* Fuco; (**C**) 9'*Z*-Fuco; (**D**) 13*Z*- and 13'*Z*-Fuco.

*Laminaria japonica* Aresch powder (Xiamen City, Fujian Province, China) was extracted with 80% (*v*/*v*) aqueous methanol, then separated by silica column chromatography, a Sephadex LH-20 column, and further purified by reversed-phase HPLC. The following amounts were obtained: Fuco (890 mg), 9'*Z*-Fuco (28.3 mg), 13*Z*- and 13'*Z*-Fuco (59.5 mg) [7]. Fuco and its stereoisomers were analyzed using an HPLC system equipped with photodiode array detector (Shimadzu LC-20AD, Shimadzu Corp., Kyoto, Japan) (Figure 2).

The purities of Fuco, 9'*Z*-Fuco and 13*Z*- and 13'*Z*-Fuco were 99.48%, 98.15%, and 96.72%, respectively. The corresponding structures illustrated in Figure 1 were established by ^1^H-NMR, ^13^C-NMR, IR and UV.

### 2.2. DPPH Radical-Scavenging Activity

**Figure 3:**
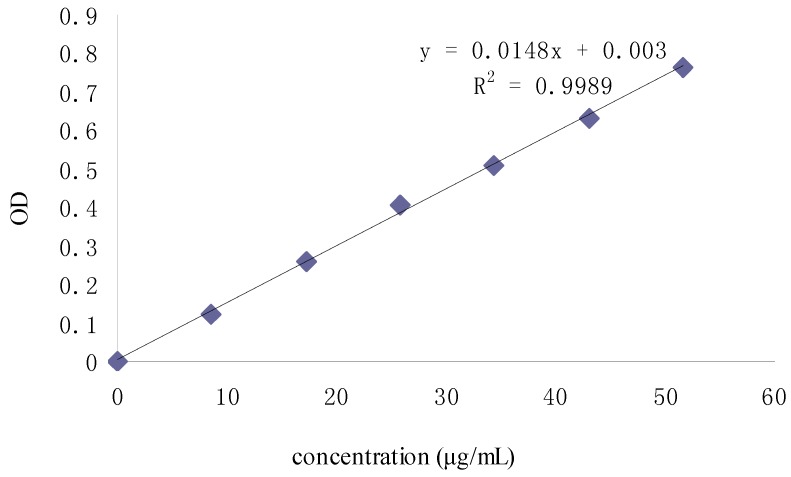
Relationship between DPPH concentration and OD.

Because of DPPH is a stable organic radical, the DPPH oxidation assay is widely used in the quantification of radical-scavenging capacity (RSC) to evaluate the antioxidant activity of biological samples [13,15,16]. The DPPH scavenging activity of three Fuco stereoisomers is presented in Figure 3. 

**Figure 4:**
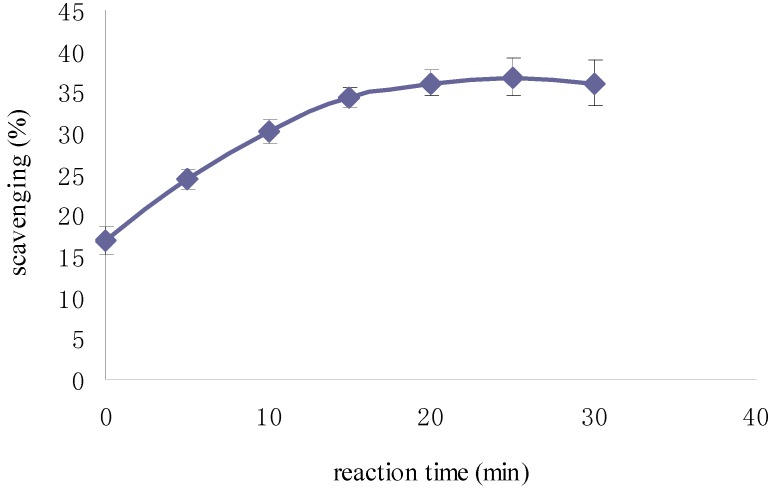
Percent scavenging of DPPH by Fuco over time.

**Figure 5:**
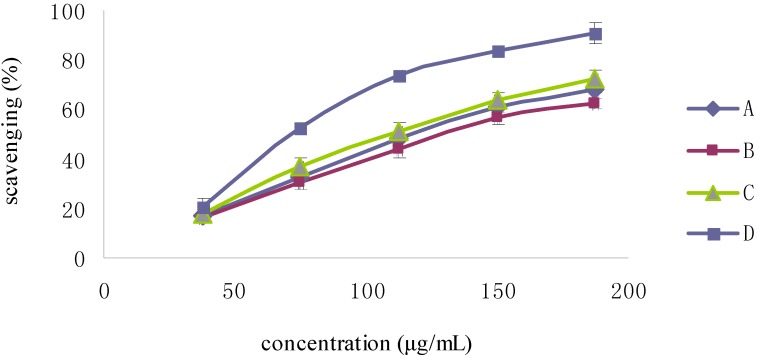
DPPH radical-scavenging activity of Fuco stereoisomers and VE. (**A**) (all-*E*)-Fuco; (**B**) 9'*Z*-Fuco; (**C**) 13*Z*- and 13'*Z*-Fuco; (**D**) VE.

**Figure 6:**
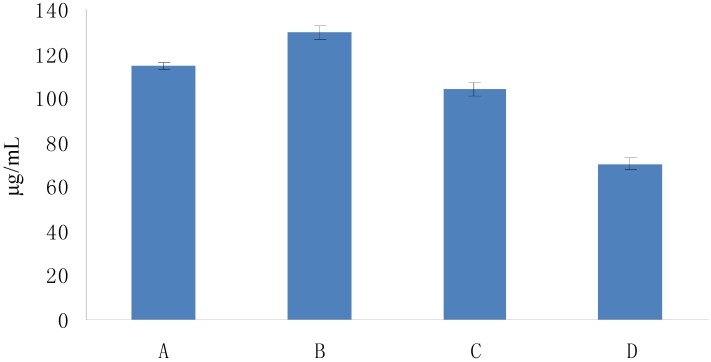
DPPH radical-scavenging activities of Fuco stereoisomers and VE as a function of their EC_50s_. (**A**) (all-*E*)-Fuco; (**B**) 9'Z-Fuco; (**C**) 13*Z*- and 13'*Z*-Fuco; (**D**) VE. The EC_50s_ were calculated from the % scavenging ability of each compound (Figure 5).

The optical density (OD) of DPPH was found to be linearly dose-dependent (8.6–51.6 μg/mL). The initial concentration of DPPH used to test the scavenging activity was 51.6 μg/mL, which corresponded to an OD of 0.822. As shown in Figure 4, the scavenging activity of Fuco first increased up to 20 min and then remained constant; moreover, it appeared dose-dependent like the scavenging activity of the three others Fuco stereoisomers (Figure 5). With regard to the EC_50_ values of DPPH radical-scavenging activities were in the order: 13*Z*- and 13'*Z*-Fuco > (all-*E*)-Fuco > 9'*Z*-Fuco; however, they were all weaker than α-tocopherol (VE) as shown in Figure 6.

### 2.3. ABTS Radical-Scavenging Activity

**Figure 7:**
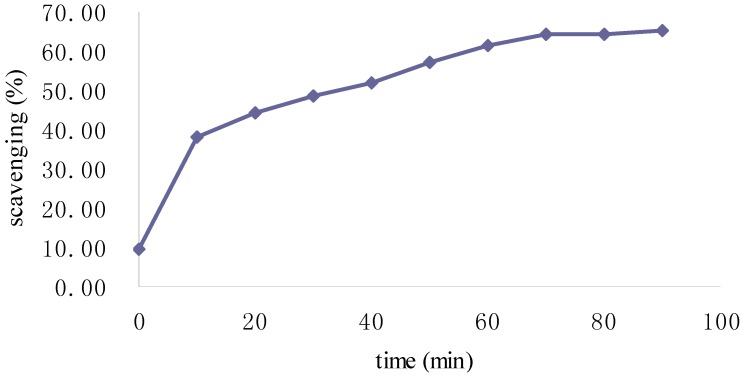
Percent scavenging of ABTS radical by Fuco over time.

The ABTS-radical-scavenging assay is a popular method for indirect determination of a compound’s antioxidant capacity [17,18]. ABTS can be oxidized by peroxy radicals to form a metastable radical that has a strong absorption between 600 nm and 750 nm. When an antioxidant is present and the ABTS radical is scavenged, the absorption decreases. The reaction of ABTS and antioxidants was correlated with reaction time; indeed, ABTS radical-scavenging activity increased rapidly within 10 min and more slowly over 70 min (Figure 7).

**Figure 8:**
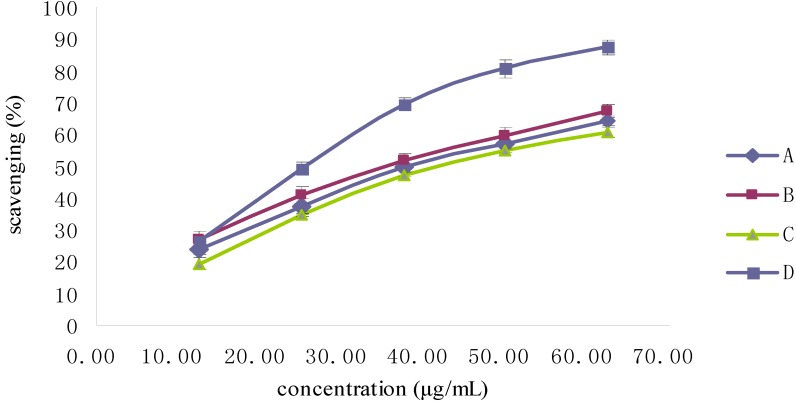
ABTS radical-scavenging activity of Fuco stereoisomers and VE. (**A**) is (all-*E*)-Fuco; (**B**) is 9'*Z*-Fuco; (**C**) is 13*Z*- and 13'*Z*-Fuco; (**D**) is VE.

**Figure 9:**
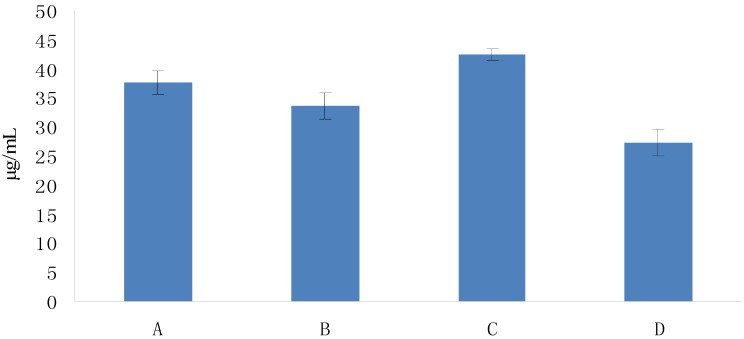
ABTS radical-scavenging activity of Fuco stereoisomers and VE as a function of their EC_50s_. (**A**) (all-*E*)-Fuco; (**B**) 9'*Z*-Fuco; (**C**) 13*Z*- and 13'*Z*-Fuco; (**D**) VE.

As illustrated in Figure 8, the ABTS radical-scavenging activities of the Fuco stereoisomers were dose-dependent in a similar way. Unlike the DPPH scavenging activity trend, 9'-*Z*-Fuco was a stronger scavenger of ABTS, with an EC_50_ value of 33.54 μg/mL, than all-*E* Fuco, while 13-*Z* and 13'-*Z*-Fuco had the lowest ABTS scavenging activities (Figure 9).

### 2.4. Hydroxyl Radical-Scavenging Activity

**Figure 10:**
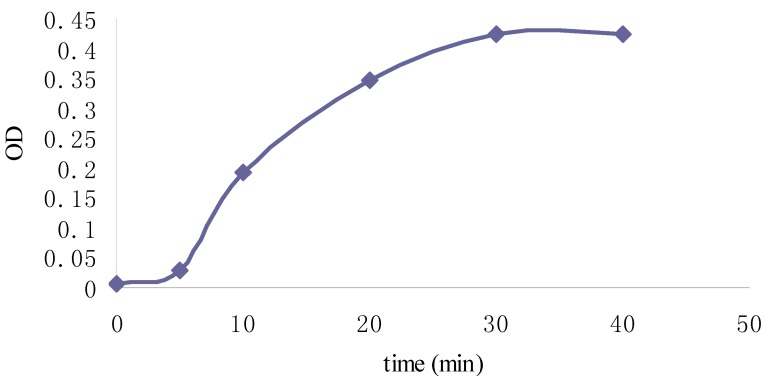
Relationship between absorbance and time for hydroxyl radical generation by Fenton’s reagent at 37 °C.

**Figure 11:**
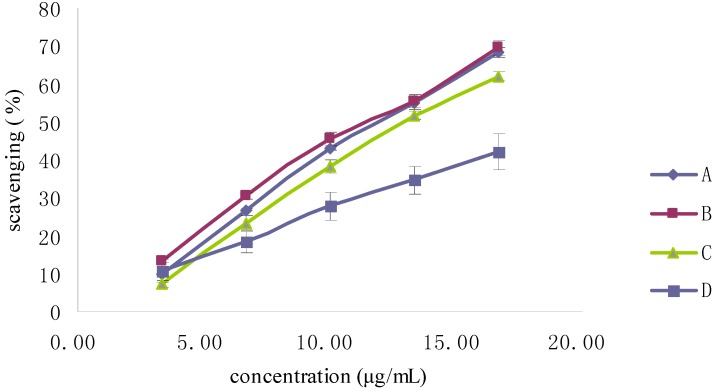
Hydroxyl radical-scavenging activity of Fuco stereoisomers and VE as measured by Fenton’s reagent. (**A**) (all-*E*)-Fuco; (**B**) 9'*Z*-Fuco; (**C**) 13*Z*- and 13'*Z*-Fuco; (**D**) VE.

**Figure 12:**
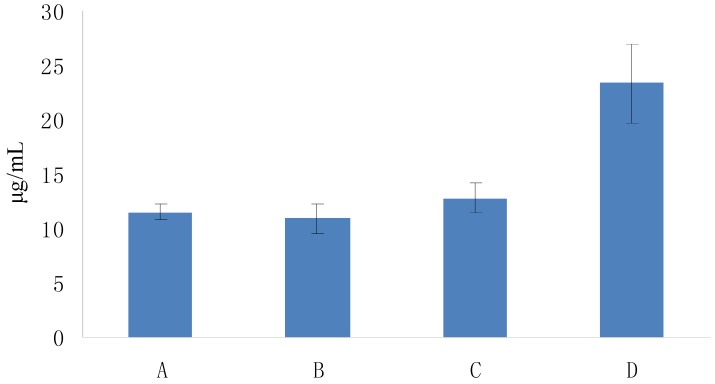
Hydroxyl radical-scavenging activity of Fuco stereoisomers and VE as a function of their EC_50s_. (**A**) (all-*E*)-Fuco; (**B**) 9'*Z*-Fuco; (**C**) 13*Z*- and 13'*Z*-Fuco; (**D**) VE.

Excessive free radicals in the body can result in oxidative damage to some organs and cause many diseases [19,20]. Fenton’s reagent can be used to generate hydroxyl radicals and the color of the Fenton system can be used to indicate the radical content. When the radicals are scavenged by antioxidants, the change in color will be proportional to the scavenged radicals. Meanwhile, the scavenging of radicals by carotenoids can be evaluated by chemiluminescence techniques. In the present study, we found that the hydroxyl radicals were inactive when the temperature was lower than 20 °C while their generation improved as the temperature increased. The reaction speed was also related to the temperature. Figure 10 shows that at a temperature of 37 °C to resemble body conditions, the hydroxyl radical-scavenging activity of Fuco increased slowly over the first 5 min, and then rapidly from 5 to 30 min. The activities of the three Fuco stereoisomers were found to be linearly dose-dependent, and were almost equivalent (Figure 11). As regards the EC_50_ values, in contrast to the case of DPPH and ABTS, Fuco and its stereoisomers presented similar scavenging activities lower than VE (Figure 12).

### 2.5. Superoxide Radical-Scavenging Activity

**Figure 13:**
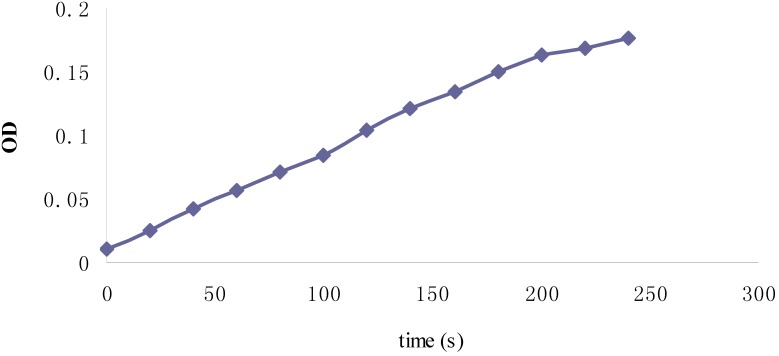
Relationship between the OD and time for determination of THDB autoxidation.

**Figure 14:**
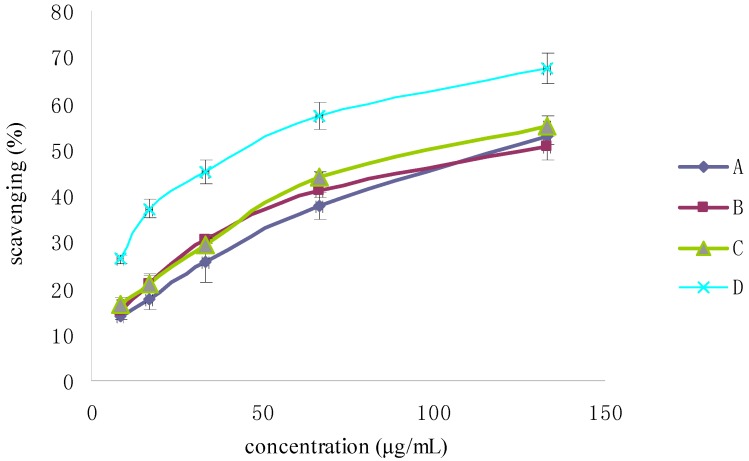
Superoxide radical-scavenging activity of Fuco stereoisomers. (**A**) (all-*E*)-Fuco; (**B**) 9'*Z*-Fuco; (**C**) 13*Z*- and 13'*Z*-Fuco; (**D**) VE.

**Figure 15:**
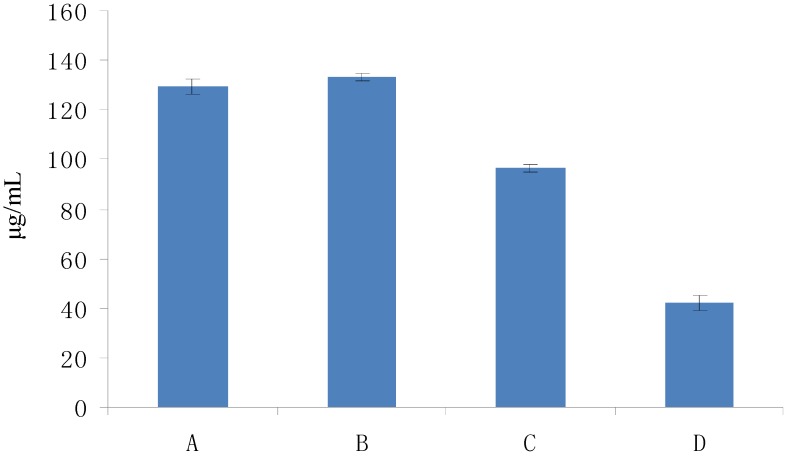
Superoxide radical-scavenging activity of Fuco stereoisomers as a function of their EC50s. (**A**) (all-*E*)-Fuco; (**B**) 9'*Z*-Fuco; (**C**) 13*Z*- and 13'*Z*-Fuco; (**D**) VE.

The 1,2,3-trihydroxybenzene (THDB) autoxidation method was used to produce and detect the superoxide radical activity. It is an indirect method widely used in the initial screening of antioxidants in the food and pharmaceutical industries. The relationship between the autoxidation and time was directly proportional over 180 s (Figure 13) and then the rate slightly diminishes from 180 to 250 s. The autoxidation of THDB stopped when VC was added. A similar dose-dependent trend of the superoxide radical scavenging activities was observed for the three Fuco stereoisomers (Figure 14). Moreover, in term of EC_50_ values, the order of superoxide radical-scavenging activity was as follows: 13*Z*- and 13'*Z*-Fuco > (all-*E*)-Fuco > 9'*Z*-Fuco (Figure 15).

## 3. Experimental

### 3.1. Materials and Chemicals

1,1-Diphenyl-2-picrylhydrazyl (DPPH), 2,2'-azinobis-3-ethylbenzothizoline-6-sulphonate (ABTS), α-tocopherol (VE) and 1,10-phenanthroline monohydrate (PHEN) were purchased from Sigma-Aldrich (Shanghai, China). 1,2,3-Trihydroxybenzene (THDB), vitamin C (VC), phosphate buffer saline (PBS), potassium persulfate, ferrous sulfate, aquae hydrogenii dioxide, Tris-HCL solution, methanol, and ethanol were purchased from Sino Pharm Chemical Reagent Co. Ltd. (Shanghai, China). All the other reagents used were of analytical grade. The radical-scavenging activity was determined by an enzyme-labeled instrument (SpectraMa MD Co., Ltd., Sunyvale, CA, USA).

### 3.2. Extraction and Isolation

The powder of *Laminaria japonica* Aresch (10 kg) was extracted with 80% aqueous methanol (50 L) at room temperature for 10 min, and then evaporated under vacuum to a something like a concrete (1.56 L). The Fuco mixture was first isolated from the methanol extract by silica column chromatography using stepwise elution with a petroleum-acetone mixture (20:1 to 1:1), and then further separated using a Sephadex LH-20 (1200 × 20 mm 27–163 μm) column saturated with 100% methanol. Finally, all-*E*-Fuco, 9'*Z*-Fuco and 13*Z*- and 13'*Z*-Fuco were successively purified by reversed-phase preparative HPLC. The purity of (all-*E*)-Fuco, 9’*Z*-Fuco and 13*Z*- and 13'*Z*-Fuco was determined on a Shimadzu LC-20AD HPLC (Shimadzu Crop.) equipped with a photodiode array detector. Ten μL of sample was injected onto an ODS C18 column (250 × 4.6 mm, 5 μm), which was equilibrated with 90% aqueous methanol as the mobile phase at a flow rate of 1.0 mL/min with detection at 450 nm.

### 3.3. DPPH Radical-Scavenging Assay

The radical-scavenging activity of 1,1-diphenyl-2-picrylhydrazyl (DPPH) was measured by the method of Nakkarike [12]. Ten μL of the sample in methanol was mixed with 0.12 mM DPPH in methanol (150 μL) and incubated in the dark at 25 °C for 30 min. Sample and DPPH solutions in methanol were used as control and blank, respectively. The optical density (OD) was measured at 517 nm, and the scavenging activity was calculated as follows:

Scavenging (%) = [1 − (A_sample_ − A_sample blank_)/A_control_] × 100%

### 3.4. ABTS Radical-Scavenging Assay

2,2-Azino-bis(3-ethylbenzothiazoline-6-sulphonic acid) (ABTS) radical solution was prepared by mixing ready-to-use ABTS aqueous solution (5 mL, 7 mM) with potassium persulfate aqueous solution (88 μL, 140 mM) at 25 °C for 15 h. Prior to decolorization, the ABTS radical solution was diluted with methanol until its OD decreased to 0.7, which then made it suitable for the ABTS radical-scavenging assay. The discoloration was initiated by mixing ABTS solution (150 μL) with the sample (10 μL) and incubating at 25 °C for 70 min. Sample and DPPH solutions in methanol were used as control and blank, respectively. The OD was measured at 734 nm at the beginning and the end of the incubation period. Finally scavenging activity was calculated as follows:

Scavenging (%) = [(*A*_control_ − *A*_sample_)/*A*_control_] × 100%

### 3.5. Hydroxyl Radical-Scavenging Assay

Hydroxyl radical was generated by the Fenton system [13,21]. The *A*_0_ reaction mixture contained PBS solution (60 μL, pH = 7.4), FeSO_4_ (60 μL, 1.5 mM), 1,10-phenanthroline monohydrate (60 μL, 1.5 mM), the methanol solution of compound (20 μL) and H_2_O_2_ (30 μL, 80 mM). The *A*_1_ reaction mixture contained PBS solution (60 μL, pH = 7.4), FeSO_4_ (60 μL, 1.5 mM), 1,10-phenanthroline monohydrate (60 μL, 1.5 mM), methanol (20 μL), and H_2_O_2_ (30 μL, 80 mM). The *A*_2_ reaction mixture contained PBS solution (60 μL, pH = 7.4), FeSO_4_ (60 μL, 1.5 mM), 1,10-phenanthroline monohydrate (60 μL, 1.5 mM) and μL methanol (20). The 96-microwell plates were filled with all the reagents except H_2_O_2_ and incubated at 37 °C for 70 min, then the reaction was started by the automatic addition of H_2_O_2_. The OD was measured at 536 nm at the start and end of the incubation period. The results were used to calculate the scavenging percent as follows:

Scavenging (*%*) = [(*A*_0_ − *A*_1_)/(*A*_2_ − *A*_1_)] × 100%

### 3.6. Superoxide Radical-Scavenging Assay

Tris-HCl (a total of 150 μL, 50 mM, pH = 8.2) was continuously added to sample (10 μL) and 1,2,3-trihydroxybenzene (THDB, 10 μL, 3 mmol/L). After 3 min, VC (10 μL, 5 mM) was added to stop the reaction (*A*_0_). Solution *A*_1_ consisted of Tris-HCl (150 μL) that was continuously added to a sample (10 μL) containing VC and 1,2,3-trihydroxybenzene. Solution *A*_2_ consisted of Tris-HCl (a total of 150 μL) that was reacted with 1,2,3-trihydroxybenzene (10 μL) for 3 min. Then VC and blank sample solution (10 μL) were added to stop the reaction. Solution *A*_3_ consisted of Tris-HCl (150 μL) that was continuously added to VC and 1,2,3-trihydroxybenzene and blank sample solution (10 μL). The OD was measured at 536 nm at the start and end of the incubation period. The results were used to calculate the scavenging percent as follows:

Scavenging (%) = [(*A*_0_ − *A*_1_)/(*A*_2_ − *A*_3_)] × 100%

## 4. Conclusions

All three Fuco stereoisomers had stronger scavenging hydroxyl radical activities than VE, but they showed weaker scavenging activities toward DPPH and superoxide radical than VE, while the radical-scavenging activities of the three Fuco stereoisomers were not remarkably different, that is, the isomeric structure differences of the Fuco stereoisomers had very little effect on their radical-scavenging activities and therefore, it is not necessary to purify the Fuco stereoisomers prior to using them as antioxidants, which can allow cost savings. Further *in vivo* studies of Fuco antioxidant activity are however needed to accelerate the use of Fuco as a natural antioxidant.
